# Frequent Prescribed Fires Can Reduce Risk of Tick-borne Diseases

**DOI:** 10.1038/s41598-019-46377-4

**Published:** 2019-07-10

**Authors:** Elizabeth R. Gleim, Galina E. Zemtsova, Roy D. Berghaus, Michael L. Levin, Mike Conner, Michael J. Yabsley

**Affiliations:** 1 Warnell School of Forestry and Natural Resources & Southeastern Cooperative Wildlife Disease Study, College of Veterinary Medicine, University of Georgia, Athens, GA 30602 USA; 2 Jones Center at Ichauway, Newton, GA 39870 USA; 3 Centers for Disease Control and Prevention, Rickettsial Zoonoses Branch, Atlanta, GA 30029 USA; 4 College of Veterinary Medicine, University of Georgia, Athens, GA 30602 USA; 5 Present Address: 8003 Fishburn Dr., Hollins University, Roanoke, VA 24020 USA

**Keywords:** Ecological epidemiology, Fire ecology, Ecological epidemiology, Fire ecology, Bacterial infection

## Abstract

Recently, a two-year study found that long-term prescribed fire significantly reduced tick abundance at sites with varying burn regimes (burned surrounded by burned areas [BB], burned surrounded by unburned areas [BUB], and unburned surrounded by burned areas [UBB]). In the current study, these ticks were tested for pathogens to more directly investigate the impacts of long-term prescribed burning on human disease risk. A total of 5,103 ticks (4,607 *Amblyomma americanum*, 76 *Amblyomma maculatum*, 383 *Ixodes scapularis*, two *Ixodes brunneus*, and 35 *Dermacentor variabilis*) were tested *for Borrelia* spp., *Rickettsia* spp., *Ehrlichia* spp., and *Anaplasma phagocytophilum*. Long-term prescribed fire did not significantly impact pathogen prevalence except that *A. americanum* from burned habitats had significantly lower prevalence of *Rickettsia* (8.7% and 4.6% for BUB and UBB sites, respectively) compared to ticks from control sites (unburned, surrounded by unburned [UBUB])(14.6%). However, during the warm season (spring/summer), encounter rates with ticks infected with pathogenic bacteria was significantly lower (98%) at burned sites than at UBUB sites. Thus, despite there being no differences in pathogen prevalence between burned and UBUB sites, risk of pathogen transmission is lower at sites subjected to long-term burning due to lower encounter rates with infected ticks.

## Introduction

There are a number of tick species of public health significance in the southeastern United States such as *Amblyomma americanum*, *Dermacentor variabilis*, *Ixodes scapularis*, and *Amblyomma maculatum*. All of these ticks are capable of transmitting one or more tick-borne pathogens. For example, *A. americanum* is the main vector of *Ehrlichia chaffeensis (*human monocytic ehrlichiosis [HME]), *Ehrlichia ewingii* (*Ehrlichia ewingii* ehrlichiosis), and Panola Mountain *Ehrlichia* (Panola Mountain ehrlichiosis). *A. americanum* is also associated with the causative agent of Southern tick-associated rash illness (STARI). Although the etiologic agent of STARI has not yet been confirmed, *Borrelia lonestari* and *Rickettsia amblyommatis* have been suggested as potential causative agents^1,2^. Other tick-borne pathogens include *Rickettsia rickettsii* (Rocky Mountain spotted fever [RMSF]) transmitted by *D. variabilis*, *Rickettsia parkeri* (*Rickettsia parkeri* rickettsiosis) transmitted by *A. maculatum*, and *Borrelia burgdorferi* (Lyme disease) and *Anaplasma phagocytophilum* (human granulocytic anaplasmosis [HGA]) both transmitted by *I. scapularis*.

The incidence of these tick-borne diseases has increased in the past several decades and several new pathogens have emerged including heartland virus, Bourbon virus, *Borrelia miyamotoi*, *Borrelia mayonii*, and *Ehrlichia muris eauclairensis*^3–5^. Thus, the need to find cost-effective, practical approaches to reducing tick-borne disease risk is more important than ever. Interestingly, Gleim *et al*.^6^ found that long-term prescribed fire significantly reduced tick abundance and altered tick species composition. However, very few studies have examined whether fire could directly impact pathogen prevalence^7^ despite some studies having indicated that habitat and ecological variables can affect pathogen dynamics^8,9^.

Importantly, prescribed fire is an especially common and necessary land management practice in fire-dependent ecosystems such as open pine forests, grasslands, and fire-maintained wetlands. Burning at different frequencies and intensities can also be an appropriate management tool in fire tolerant hardwood forests^10,11^. In all of these ecosystems, prescribed fire is typically used to suppress undesirable woody vegetation, stimulate herbaceous growth of the understory, and facilitate seed germination. This reduces fuel loads and wildfire risk, provides enhanced habitat for wildlife, and increases overall ecosystem health^12,13^.

To follow-up on our finding that long-term prescribed fire significantly reduced tick abundance^6^, the current study tested the ticks collected in that previous study for common tick-borne pathogens to investigate how prescribed fire may affect pathogen dynamics. This would allow us to more definitively determine the impacts of long-term prescribed fire on human disease risk. This study also provided a basic understanding of tick-borne pathogen dynamics in geographically and ecologically unique regions of the southeastern United States in which little was known.

## Results

In total 5,103 ticks were tested for one or more pathogens (4,607 *A. americanum*, 76 *A. maculatum*, 383 *I. scapularis*, two *I. brunneus*, and 35 *D. variabilis*). Burn treatments were found to have a significant effect on the minimum infection prevalence of *Rickettsia* spp. in *A. americanum* (p = 0.026) with BUB, UBB, and UBUB having 8.7% (19/219), 4.4% (16/361), and 16.7% (584/3490) prevalence, respectively (Table 1). Importantly, minimum infection prevalence is the most conservative estimate of pathogen prevalence and it is possible that the prevalence is higher, particularly for pathogens that occur at a higher prevalence. The BB study site only had a single *A. americanum*, which was positive. Burn treatment was not found to have a significant effect on the prevalence of any other pathogens.

**Table 1:** Results of *Rickettsia* spp. testing by burn treatment (burned surrounded by burned [BB], burned surrounded by unburned [BUB], unburned surrounded by burned [UBB], and unburned surrounded by unburned [UBUB]).

Tick species	Treatment	Total*	Adult	Nymph**	Larvae**	Organisms Detected^Ɨ^
*A. americanum*	BB	1/1 (100)	1/1 (100)	—	—	1 *R. amblyommatis*
	BUB	19/219 (8.7)	9/15 (60.0)	4/21 (19.0)	6/183 (3.3)	16 *R. amblyommatis*
	UBB	16/350 (4.6)	8/13 (61.5)	2/16 (12.5)	6/321 (1.9)	13 *R. amblyommatis*
	UBUB	589/4037 (14.6)	280/441 (63.5)	212/1152 (18.4)	97/2444 (4.0)	412 *R. amblyommatis*
						1 *Rickettsia* sp. (95%, 98%, DQ092218)
*A. maculatum*	BB	7/37 (18.9)	7/36 (19.4)	0/1 (0)	—	1 *R. amblyommatis*
						1 *R. parkeri*
	BUB	4/30 (13.3)	4/29 (13.8)	0/1 (0)	—	
	UBB	2/6 (33.3)	2/5 (40.0)	0/1 (0)	—	
	UBUB	1/3 (33.3)	1/3 (33.3)	—	—	1 *R. amblyommatis*
*I. scapularis*	BB	0/2 (0)	0/2 (2)	—	—	
	BUB	9/37 (24.3)	9/17 (52.9)	—	0/20 (0)	1 *R. cooleyi*
						6 *Rickettsia* sp. TR-39
	UBB	42/100 (42.0)	42/99 (42.4)	0/1 (0)	—	1 *R. amblyommatis*
						3 *R. monacensis*
						17 *Rickettsia* sp. TR-39
						2 *Rickettsia* sp. (96–98%, KC003474)
						1 *Rickettsia* sp. (99%, JN190456)
	UBUB	68/132 (51.5)	67/128 (52.3)	1/4 (25.0)	—	2 *R. cooleyi*
						1 *R. monacensis*
						24 *Rickettsia* sp. TR-39
						4 *Rickettsia* sp. TX140 (99–100%, EF689739)
						1 *Rickettsia* sp. (84%, KC003474)
						1 *Rickettsia* sp. (97%, EU283838)
*D. variabilis*	BB	—	—	—	—	
	BUB	2/8 (25.0)	2/8 (25.0)	—	—	1 *Rickettsia* sp. TR-39
						1 *R. amblyommatis* (79%, GQ302891)
	UBB	1/9 (11.1)	1/9 (11.1)	—	—	1 *R. rhipicephali*
						1 *R. amblyommatis*
	UBUB	7/18 (38.9)	7/18 (38.9)			

*Number of infected ticks (or pools when applicable) over total ticks tested (percent positive).

**For nymphs and larvae, indicates minimum infection prevalence.

^Ɨ^For 2010 & 2011, some positive samples were unsequenceable or were not sent for sequencing. For 2011 only, all samples that were sequenced had a minimum of 95% identity unless otherwise noted.

In *A. maculatum* adults, 19.2% (n = 73) were positive for *Rickettsia* spp. Three of the 14 positives could be identified to species, of which one was positive for *R. parkeri* (1.4%) (Table 1). In *A. americanum*, overall *Rickettsia* spp. prevalence ranged between 3.7% and 63.4% based on the life stage. Among the *Rickettsia* identified from *A. americanum* (n = 444) (Table 1), 95% were identified as *R. amblyommatis* (previously *Candidatus R. amblyommii*). In *I. scapularis* adults, 48% were positive for *Rickettsia* spp. with *Rickettsia* sp. TR-39 being the most commonly identified. Other endosymbionts identified in *I. scapularis* included *Rickettsia cooleyi*, *Rickettsia monacensis*, *R. amblyommatis*, and *Rickettsia* sp. TX140 (Table 1). In *D. variabilis* adults, 28.6% were positive for *Rickettsia* spp. and although a number of endosymbionts were detected (*Rickettsia rhipicephali*, *R. amblyommatis*, and *Rickettsia* sp. TR-39), importantly, no *R. rickettsii* was detected.

Regarding *Ehrlichia* spp. (Table 2), in 2011, only *A. americanum* were tested for *Ehrlichia* spp., whereas in 2010, *A. americanum, A. maculatum*, and *D. variabilis* were tested. In *A. americanum* adults, prevalence of 0.6%, 4.5%, and 1.0% were detected for *E. chaffeensis*, *E. ewingii* (n = 470 for both), and Panola Mountain *Ehrlichia* sp. (n = 293), respectively. Among *A. americanum* nymphs, minimum infection prevalences of 0.2% and 0.3% (n = 1189 & 1086, respectively) were detected for *E. chaffeensis* and Panola Mountain *Ehrlichia* sp. in both years. In 2010, two *D. variabilis* (3.8%, n = 26) were positive for *E. ewingii* or the Panola Mountain *Ehrlichia* sp. respectively. With the exception of a single *E. ewingii* positive tick from a BUB site, all other *Ehrlichia* spp. positive ticks originated from UBUB sites. All *A. maculatum* (n = 57) and *A. americanum* larvae (n = 1400) were negative for all three *Ehrlichia* spp.

**Table 2:** Results of *Ehrlichia* spp. testing by burn treatment (burned surrounded by burned [BB], burned surrounded by unburned [BUB], unburned surrounded by burned [UBB], and unburned surrounded by unburned [UBUB]).

Pathogen		*A. maculatum*		*A. americanum*			*D. variabilis*	Total**
		adult	nymph**	adult	nymph**	larvae**	adult	
*E. chaffeensis*	BB	0/30 (0)*	0/1 (0)	0/1 (0)	—	—	—	0/32 (0)
	BUB	0/24 (0)	—	0/15 (0)	0/21 (0)	0/83 (0)	0/6 (0)	0/150 (0)
	UBB	0/3 (0)	—	0/13 (0)	0/16 (0)	0/201 (0)	0/7 (0)	0/240 (0)
	UBUB	—	—	3/441 (0.7)	2/1152 (0.2)	0/1116 (0)	0/13 (0)	5/2724 (0.2)
	**Total**	**0/57 (0)**	**0/1 (0)**	**3/470 (0.6)**	**2/1189 (0.2)**	**0/1400 (0)**	**0/26 (0)**	**5/3146 (0.2)**
*E. ewingii*	BB	0/30 (0)	0/1 (0)	0/1 (0)	—	—	—	0/32 (0)
	BUB	0/24 (0)	—	0/15 (0)	0/21 (0)	0/83 (0)	1/6 (16.7)	1/150 (0.7)
	UBB	0/3 (0)	—	0/13 (0)	0/16 (0)	0/201 (0)	0/7 (0)	0/240 (0)
	UBUB	—	—	21/441 (4.8)	0/1152 (0)	0/1116 (0)	0/13 (0)	21/2724 (0.8)
	**Total**	**0/57 (0)**	**0/1 (0)**	**21/470 (4.5)**	**0/1189 (0)**	**0/1400 (0)**	**1/26 (3.8)**	**22/3146 (0.7)**
Panola Mountain *Ehrlichia*	BB	0/30 (0)	0/1 (0)	—	—	—	0	0/32 (0)
	BUB	0/24 (0)	—	0/10 (0)	0/4 (0)	0/83 (0)	0/6 (0)	0/150 (0)
	UBB	0/3 (0)	—	0/9 (0)	0/12 (0)	0/201 (0)	0/7 (0)	0/240 (0)
	UBUB	—	—	3/274 (1.1)	3/1070 (0.3)	0/1116 (0)	1/13 (7.7)	7/2724 (0.2)
	**Total**	**0/57 (0)**	**0/1 (0)**	**3/293 (1.0)**	**3/1086 (0.3)**	**0/1400 (0)**	**1/26 (3.8)**	**7/3146 (0.2)**

*Number of infected ticks (or pools when applicable) over total ticks tested (percent positive).

**Minimum infection prevalence.

*Borrelia* infections were rare and none were detected in *A. maculatum* (n = 57), *I. scapularis* (n = 383), or *I. brunneus* (n = 2). *B. lonestari* was detected in *A. americanum* adults (n = 470), nymphs (n = 1189), and larvae (n = 1400) at 0.6%, 0.7%, and 1.1% prevalences, respectively (Table 3). Similarly, *A. phagocytophilum* was rare with only 1.1% of adult *I. scapularis* being positive (Table 4). None of the *I. scapularis* nymphs or *I. brunneus* were positive.

**Table 3:** Results of *Borrelia lonestari* testing by burn treatment (burned surrounded by burned [BB], burned surrounded by unburned [BUB], unburned surrounded by burned [UBB], and unburned surrounded by unburned [UBUB]).

	*A. americanum*			Total
	adult	nymph**	larvae**	
BB	0/1 (0)*	—	—	**0/1 (0)**
BUB	1/15 (6.7)	0/21 (0)	1/83 (0.1)	**2/120 (1.7)**
UBB	0/13 (0)	0/16 (0)	0/201 (0)	**0/233 (0)**
UBUB	2/441 (0.4)	9/1152 (1.0)	14/1116 (1.2)	**25/2710 (0.9)**
Total	**3/470 (0.6)**	**9/1189 (0.7)**	**15/1400 (1.1)**	**27/3064 (0.9)**

*Number of infected ticks (or pools when applicable) over total ticks tested (percent positive).

**Minimum infection prevalence.

**Table 4:** Results of *Anaplasma phagocytophilum* testing by burn treatment (burned surrounded by burned [BB], burned surrounded by unburned [BUB], unburned surrounded by burned [UBB], and unburned surrounded by unburned [UBUB]).

	*I. scapularis*		*I. brunneus*	Total
	adult	nymph	nymph	
BB	0/5 (0)*	—	—	**0/5 (0)**
BUB	1/30 (3.3)	—	—	**1/30 (3.3)**
UBB	2/141 (1.4)	0/1 (0)	0/2 (0)	**2/144 (1.4)**
UBUB	1/192 (0.5)	0/12 (0)	—	**1/204 (0.5)**
**Total**	**4/368 (1.1)**	**0/13 (0)**	**0/2 (0)**	**4/383 (1.0)**

Note that two of our *A. phagocytophilum* positive sequences were evaluated and determined to be 99.7% identical to variants detected in cervids.

*Number of infected ticks over total ticks tested (percent positive).

**Figure 1:**
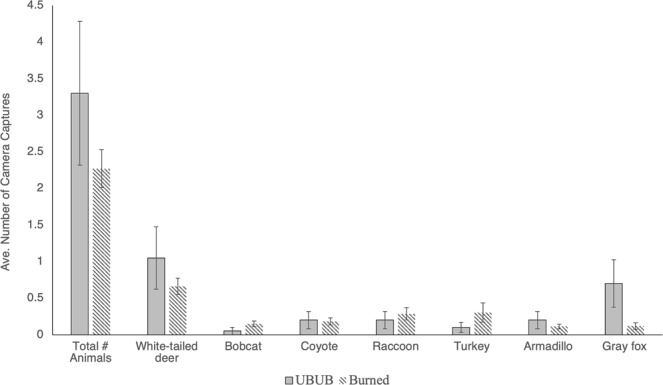
Average total number of individuals and average number of each individual wildlife species (+/− standard error) considered in the negative binomial models per site per quarter at unburned, unburned sites (UBUB) and burned sites. Importantly, none of the host variables were found to significantly impact the number of ticks encountered per hour that were infected with any bacteria or pathogenic bacteria. Therefore, no host variables were included in the final negative binomial models.

**Figure 2:**
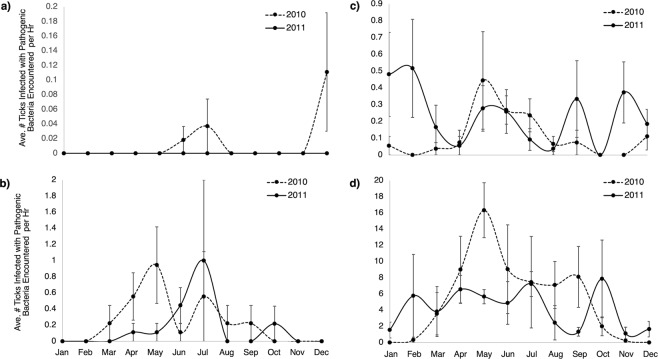
Average number of ticks encountered per hour (+/−standard error) that were infected with pathogenic bacteria in burned (**a**) and unburned, unburned sites (UBUB) (**b**) and any bacteria in burned (**c**) and UBUB sites (**d**).

Based on the negative binomial regression models, the likelihood of encountering a tick infected with pathogenic bacteria or any bacteria was significantly higher at UBUB sites than at burned sites (Tables 5 and 6). Interestingly, no wildlife species were found to impact encounter rates with ticks with pathogenic or any bacteria (Tables 5 and 6, Fig. 1). Over the two-year sampling period, the peak average (+/−SE) encounter rate with ticks infected with pathogenic bacteria at burned sites was 0.11 +/− 0.08 infected ticks per hour with an overall average of only 0.007 +/− 0.005 infected ticks per hour (Fig. 2). In contrast, at UBUB sites, the peak average encounter rate with ticks infected with pathogenic bacteria was 1.0 +/− 1.0 infected ticks per hour with an overall average of 0.20 +/− 0.06 infected ticks per hour.

**Table 5:** Results of the negative binomial regression which examined the impacts of long-term prescribed burning, year, quarter, and wildlife occurrence on the number of ticks encountered per hour that were infected with pathogenic bacteria.

	Coefficient (SE)	RR (95% CI)	P
Any Burning (vs. No Burning*)	−0.65 (1.17)	ND	0.579
Season (Warm [Spring/Summer] vs Cool* [Fall/Winter])	2.51 (1.26)	ND	0.047
Any Burn X Season	−3.43 (1.65)	ND	0.037
Constant	−3.50 (0.92)	NA	<0.001
ln(effort)	1 (exposure)		

SE = Standard error. RR = Relative rate. ND = Not determined; RR is not given because it depends on the interacting variable. NA = Not applicable.

*Indicates the reference category.

**Table 6:** Results of the negative binomial regression which examined the impacts of long-term prescribed burning, year, quarter, and wildlife occurrence on the number of ticks encountered per hour that were infected with any bacteria.

	Coefficient (SE)	RR (95% CI)	P
Any Burning (vs. No Burning*)	−2.09 (0.50)	ND	<0.001
Season (Warm [Spring/Summer] vs Cool* [Fall/Winter])	1.54 (0.52)	ND	0.003
Any Burn X Season	−1.58 (0.62)	ND	0.011
Year (2011 vs. 2010*)	0.44 (0.19)	1.55 (1.06, 2.27)	0.024
Constant	−882 (390)	NA	0.024
ln(effort)	1 (exposure)		

SE = Standard error. RR = Relative rate. ND = Not determined; RR is not given because it depends on the interacting variable. NA = Not applicable.

*Indicates the reference category.

In both negative binomial models there was a significant interaction between the effects of burning and season (p = 0.037 for the “pathogenic” model; p = 0.011 for the “any bacteria” model). In the case of the pathogenic model, it was found that encounter rates with pathogenic ticks were 98% lower in the burned sites as compared to UBUB sites during the warm season (spring/summer) (RR [95% CI] = 0.017 [0.003, 0.087]; p < 0.001), but were not significantly lower during the cool season (fall/winter) (RR [95% CI] = 0.52 [0.05, 5.17]; p = 0.579), reflecting the general decrease in pathogenic tick activity at all sites during that season (Fig. 2). When examining the “any bacteria” model, encounter rates with ticks infected with any bacteria were 88% lower in the burned sites versus UBUB sites in the cool season (RR [95% CI] = 0.12 [0.05, 0.33]; p < 0.001) and 97.5% lower in the warm season (RR [95% CI] = 0.025 [0.011, 0.061]; p < 0.001).

## Discussion

This study was the first large scale tick-borne pathogen survey performed in southwestern Georgia and northwestern Florida, thus providing valuable insight into the tick-borne pathogen dynamics in that region. Pathogen prevalences were similar to what has been reported in other parts of Georgia and neighboring states^14–16^. Although different types of assays (traditional, nested, and real-time PCR) were used to test for pathogens and some assays changed from the first to second year of testing, this still provided valuable insight into tick-borne pathogen dynamics in an under-studied region and in under-studied ecosystem types (pine and mixed pine forests). Furthermore, because any differences in assay types would have been distributed across ticks from all sites, we do not feel that this impacted the statistical comparisons between burn treatments.

Of note were the relatively high prevalences and high diversity of *Rickettsia* spp. endosymbionts in *D. variabilis* (28.6%) and *I. scapularis* (44%) and the failure to detect the known pathogenic bacteria, *R. rickettsii* in *D. variabilis*. In the case of *D. variabilis*, the low number of ticks tested may have resulted in an inaccurate portrayal of *Rickettsia* spp. diversity and prevalence, particularly as it related to *R. rickettsii*. However, previous studies have typically found much lower Spotted Fever Group (SFG) *Rickettsia* prevalence in *D. variabilis* than what was detected in this study^17,18^. For example, in Maryland prevalences of 3.8%^19^ for SFG *Rickettsia* were documented while a study in Ohio documented a 0.2% prevalence^20^. In a neighboring state to Georgia, Loving *et al*.^21^ reported a 2.4–3.9% prevalence of SFG *Rickettsia* over a three year period in South Carolina. Interestingly, it has been hypothesized that a non-pathogenic SFG *Rickettsia*, *R. peacockii*, can inhibit transovarial transmission of *R. rickettsii*, thus limiting its distribution in some areas^22,23^. Indeed, Dergousoff *et al*.^24^ found a 76% prevalence of *R. peacockii* in *D. variabilis* and *D. andersoni* in Canada, while finding no *R. rickettsii*. It is difficult to draw a conclusion in the current study due to the low sample size of *D. variabilis*. However, the high prevalence and diversity of non-pathogenic *Rickettsia* spp. observed in *D. variabilis* in this study may be playing a role in driving *R. rickettsii* dynamics within the region.

The absence of *B. burgdorferi* agrees with other studies of this pathogen in the southeastern United States which note that prevalences are significantly lower compared to prevalences in the northeastern and Midwestern US^25,26^. The cause of this disparity is not entirely understood; however, it is suspected that differences in host ecology and/or tick questing or feeding behavior may play a role^27,28^. Recently, however, there have been increased/first detections of *B. burgdorferi*-infected *I. scapularis* in some southeastern states (i.e., Kentucky and Tennessee) so continued surveillance is warranted^29,30^.

We also found a low prevalence of *A. phagocytophilum* which is similar to what past studies performed in the southeastern United States have found. For example, Fang *et al*.^31^ tested *I. scapularis* from 15 sites throughout the Lower Coastal Plain region in South Carolina, Georgia, and Florida and found prevalences ranging between 0–4.1% with the exception of Jekyll Island, Georgia which had a prevalence of 20%. Other studies in Georgia have found low prevalences and in many cases *A. phagocytophilum* was not detected^32^. We evaluated two of our *A. phagocytophilum* positive sequences and determined that they were 99.7% identical to variants detected in cervids (data not shown). Thus, these *A. phagocytophilum* do not appear to be the AP-ha variant, associated with human disease^33^ but rather are likely a white-tailed deer variant. Based on limited surveillance, white-tailed deer variants are commonly detected in Georgia^34^ but to date have not been found to cause disease in humans^33^.

While a number of studies have looked at the impacts of prescribed fire on tick abundance^6,35–43^, only a single study recently conducted after a wildfire in California has examined the impacts of fire on tick-borne pathogen prevalence^7^. Unfortunately, due to low prevalence of non-pathogenic *Borrelia* spp. (the only pathogens detected) in the California study, the impacts of the wildfire on pathogen prevalence was unclear. Meanwhile, no study has evaluated the impacts of long-term prescribed fire on tick-borne pathogen prevalence. Importantly, if we are to better understand the impact of prescribed fire on disease risk, we must understand fire impacts on both tick abundance and pathogen prevalence (and therefore the encounter rates with infected ticks in an area).

Long-term prescribed fire significantly reduced the chance of encountering a tick infected with pathogenic bacteria but did not affect the prevalence of pathogenic bacteria. Thus, in this particular system, the reduction in disease risk can be attributed to the overall reduction in ticks and not a reduction in pathogenic bacteria prevalence itself. Interestingly, it did not appear that wildlife host occurrence played a role in the encounter rates of ticks infected with pathogenic bacteria or any bacteria. However, considering the relatively low number of ticks with pathogenic bacteria in conjunction with the relatively few sightings of any given wildlife species during each trail camera survey, this study was limited in its ability to evaluate how wildlife impacted the density of ticks with pathogenic bacteria.

While burning was not found to affect prevalence of pathogenic bacteria, we did find that UBUB sites had higher prevalences of *Rickettsia* spp. in *A. americanum* than in burned sites. This seems to indicate that burning not only reduces the abundance of *A. americanum*^6^ but also alters or interrupts transmission and maintenance of at least some bacteria. While these *Rickettsia* spp. are generally thought to be non-pathogenic, there have been a small number of studies and case reports that suggest that these species may occasionally cause disease. For example, one case report tied *R. montanensis* to mild illness^44^, while several studies have speculated that *R. amblyommatis* may occasionally cause disease^45,46^.

It could be argued that *Rickettsia* spp. were the only species affected due to the fact that *A. americanum* (which is known for carrying high prevalences of *Rickettsia* spp.) dominated in UBUB sites, whereas *A. americanum* made up a small proportion of the ticks collected at burned sites^6^. However, *A. americanum* was still captured in sufficient numbers at UBB and BUB sites making it unlikely that differences in capture rates would have resulted in significantly different prevalences. It is possible, however, that only *Rickettsia* spp. were affected in part due to its relatively high prevalence compared to other tick-borne bacteria. Thus, the ubiquity of *Rickettsia* spp. may have lent itself to reflecting changes in pathogen dynamics more so than other bacteria which occur at much lower prevalences.

There are several hypotheses regarding why these burned sites had significantly lower *Rickettsia* spp. prevalences than our UBUB sites: (1) the decrease in prevalence at burned sites may be caused by the significant, long-term reduction of tick populations observed at burned study sites^6^. Although *Rickettsia* spp. are primarily maintained via transovarial transmission, wildlife hosts may play a role in transmission as well. Indeed, the fact that *Rickettsia* spp. prevalence increased from one life stage to the next in our data indicates that wildlife hosts do play a role in *Rickettsia* spp. transmission. Thus, the long-term reductions in ticks could lead to reduced transmission and overall lower ubiquity of this bacteria in the enzootic cycle, thus lowering overall *Rickettsia* spp. prevalence. (2) Long-term prescribed fire also alters habitat which would directly impact the type of hosts present within these ecosystems. While it is unclear whether this may actually impact pathogen prevalence, it is possible that changes in host dynamics also contribute towards altered pathogen dynamics in these burned areas. In particular, white-tailed deer are known to prefer habitat associated with UBUB forests^47^. Although no studies have evaluated the potential for white-tailed deer to become bacteremic with *R. amblyommatis*, other *Rickettsia* spp. have been detected in the blood of cervids^48–51^. (3) Because long-term prescribed fire alters habitat, it also affects the microclimate at these sites. Generally speaking, sites subjected to long-term prescribed fire have a diverse understory, minimal to no midstory, and a semi-open pine canopy. This forest structure creates a harsh microclimate for some tick species as they would experience higher temperatures, increased wind, and thus lower humidity. While previous studies have found that this habitat is responsible for reduced survival of *A. americanum* in burned habitats^43^, it may also affect the ability of these ticks to maintain *Rickettsia* spp. infection. Indeed, under laboratory conditions, *Rickettsia* spp. responded to changes in temperatures with some species being unable to grow at extreme temperatures that could be feasibly reached in direct sunlight in hot climates such as southern Georgia and northwestern Florida^52^.

## Conclusion

These findings have exciting implications for public health as it appears that prescribed fire, when performed on a regular basis (regardless of burn regime), significantly reduces encounter rates with ticks infected with pathogenic bacteria. Specifically, during the warm season when ticks are most active, the encounter rates with ticks infected with pathogenic bacteria was 98% lower in burned versus UBUB sites. While these reduced encounter rates are primarily due to overall reductions in tick abundance at sites subjected to long-term prescribed fire, regular prescribed fire may also be capable of reducing the transmission of certain tick-borne bacteria. Further investigation into how long-term prescribed fire might affect pathogenic bacteria such as *B. burgdorferi* in the northeastern U.S. is warranted.

Importantly, Gleim *et al*.^6^ did not observe temporary reductions in tick populations after prescribed fire but rather sustained reductions in tick abundance for the duration of the two-year study. Of note, small-scale, singular burns would not achieve these results and in fact could cause an increased number of ticks in an area due to influx of hosts using the early successional habitat^7,40^. Instead, Gleim *et al*.^43^ found that the forest structure achieved in this study (i.e. lack of mid-story and semi-open canopy) through regular, long-term prescribed fires resulted in a drier microclimate at ground-level which was critical to achieving the sustained tick reductions observed by Gleim *et al*.^6^ and therefore lower encounter rates of infected ticks documented in our current study. Because all of our burned sites had been burned on a regular basis for a minimum of ten years, further research needs to occur to determine how long regular burns would have to occur in order to achieve the results observed in this study. Additionally, the particular habitat and microclimatic conditions that are required for the results observed in this study seem to imply that the ability of fire to reduce tick populations and disease risk may vary depending on ecosystem-type and the management objectives of the prescribed fire (i.e. the extent at which forest structure is altered). Thus, similar studies need to be conducted in different ecosystems and regions of the country to determine whether long-term prescribed burning could have effects similar to those observed in the current study on different pathogens and/or within different ecosystems.

## Materials and Methods

### Study area

The sites for this study were located in southwestern Georgia and northwestern Florida which is dominated by pine and mixed-pine forests, as well as agriculture. Prescribed burning is commonly used throughout the region to maintain open pine forests including longleaf pine ecosystems. Twenty-one sites were selected based on having had a long-term (ten or more years) presence or absence of prescribed fire. To account for prescribed fire management both within the sites and immediately surrounding the sites, each site was further categorized as being (1) burned surrounded by burned areas (BB), (2) burned surrounded by unburned areas (BUB), (3) unburned surrounded by burned areas (UBB) and (4) unburned surrounded by unburned areas (UBUB) (i.e. a control). Importantly, “burned” or “unburned” in these site definitions means burned long-term or unburned long-term, respectively. For burned sites, burning had historically occurred every 2 to 4 years during the dormant season with all sites being burned during the study based on schedules determined by their respective land managers. More details on collection sites are available in Gleim *et al*.^6^.

### Tick collections, identification, and host monitoring

Tick collection, identification, and host monitoring methods were previously described in Gleim *et al*.^6^. Briefly, ticks were collected via flagging each site monthly for two years (January 2010 to December 2011). Wildlife host occurrence was monitored at each site quarterly (with the exception of winter 2010) using passive, three day trail camera surveys (Cuddeback Capture, Green Bay, WI). No permits or Institutional Animal Care and Use Committee approval are required for passive trail camera surveys or collection of ticks. Permissions to work on public and private lands were given by the Georgia Department of Natural Resources and land owners respectively.

### Pathogen testing

DNA extractions of ticks were performed using a Qiagen DNeasy blood and tissue kit (Germantown, MD) per the manufacturer’s instructions. All adult ticks were individually extracted and tested. All DNA was stored at −20 °C until PCR testing. Nymphs of the same species and from the same site and date were extracted in pools of five. For larvae, a maximum of 100 larvae of the same species and from the same site and date were extracted in pools of 20 with each pool being from a different clutch if possible. Because the same sites were sampled in 2011, *A. americanum* nymphs collected in 2011 (in pools of 5 from the same site and date) were randomly selected for testing from different sites and days. For instances in which pools were tested for pathogens, the minimum infection prevalence was calculated in which each positive pool was counted as a single positive tick. Thus, the minimum infection prevalence provides the most conservative estimate of actual pathogen prevalence.

In 2010, all *Amblyomma* spp. and *D. variabilis* were screened for *Rickettsia* spp., *E. chaffeensis* and *E. ewingii* using a multiplex quantitative polymerase chain reaction (qPCR) targeting the 17 kDa gene of *Rickettsia* spp. and the 16S rRNA gene for both *Ehrlichia* species using primers Ech16S-17/Ech16S-99, and probe Ech16S-FAM, Ech16S-17/Ech16S-99, and probe EEW16S HEX, and R17K135F/R17K249R, and probe R17KBC^53^. To identify *Rickettsia* spp., all samples positive from the multiplex assay were analyzed using a restriction fragment-length polymorphism (RFLP) assay targeting the rOmpA gene using primers RR190.70 and RR190.701R^54^ followed by the restriction enzymes *RsaI* and *PstI*^55^.

In 2011, all tick species were tested for *Rickettsia* spp. utilizing a nested PCR targeting the 17 kDa gene using 17 kD5/17kD3 primers for the primary reaction and 17 kD1/17 kD2 primers for the secondary reaction^56^. Approximately half of the *Rickettsia* spp. positive samples (a total of 350 ticks/pools of ticks which in total included 1,489 ticks) from 2011 were purified using a QIAquick gel extraction kit and sequenced at the Georgia Genomics Facility (Athens, GA). All *A. americanum* from 2011 were tested for *E. chaffeensis* using nested PCR targeting the 16S rRNA gene using primers ECC/ECB for the primary reaction and HE1/HE3 for the secondary reaction^57^. Similarly, *E. ewingii* was tested for using a nested PCR targeting the 16S rRNA gene using primers ECC/ECB for the primary reaction and HE3/EE72 for the secondary reaction^57,58^.

Finally, in 2010 only, Panola Mountain *Ehrlichia* (PME) was tested for using a nested PCR targeting the citrate synthase (gltA) gene using primers CS-185F/CS-777R for the primary reaction and CS-214F/CS-619R for the secondary reaction^59^. The results of *A. maculatum* PME testing were included in a larger statewide data set published by Loftis *et al*.^60^. Because our data in Loftis *et al*.^60^ did not differentiate among other ticks tested from various sources in Georgia, we have included the *A. maculatum* PME data here.

All *A. americanum* and *Ixodes* spp. were tested for *Borrelia* spp. using a nested PCR protocol targeting the *fla*B gene using FLALL/FLARL primers for the primary reactions and FLALS/FLARS primers for the secondary reactions^61^. All *Ixodes* spp. were tested for *Anaplasma* spp. using a PCR assay targeting the *msp2* gene using msp2-3f/msp2-3r primers^62^. All positive samples were identified by bi-directional sequencing at the Centers for Disease Control and Prevention (Atlanta, GA).

All DNA extraction, primary, and secondary reactions were run in separate areas designated for that purpose. A negative control (i.e., water) was included with each batch of extractions and PCR reactions. Appropriate positive controls were included in all batches of PCR.

### Statistics

Generalized estimating equations (GEE) logistic regression models were used to examine whether long-term prescribed fire impacted the prevalence of the following pathogens within their respective tick species: *Rickettsia* spp. in *A. americanum*, *Rickettsia* spp. in *A. maculatum*, *Rickettsia* spp. in *I. scapularis*, *B. lonestari* in *A. americanum*, and *A. phagocytophilum* in *I. scapularis*. *Ehrlichia* spp. were not examined due to the fact that there was only a single tick positive for *Ehrlichia* in burned sites. Positive pools of ticks were counted as 1 positive tick.

To further understand how host occurrence and long-term prescribed fire was impacting disease risk and pathogen dynamics, GEE negative binomial regression models were used to examine (1) the encounter rates of ticks positive for any type of pathogenic bacteria (e.g. *E. chaffeensis*, *E. ewingii*, Panola Mountain *Ehrlichia*, *A. phagocytophilum*, and *R. parkeri*) and (2) the encounter rates of ticks positive for any type of bacteria. In both models, a single pool of larvae or nymphs positive for a particular pathogen was counted as 1 positive tick. Each model evaluated the impacts of burning, season, year, the number of times each wildlife species was captured on the camera (wildlife species which were observed in at least 8 separate surveys over the course of the study were considered in the model which included white-tailed deer, bobcats [*Lynx rufus*], coyotes [*Canis latrans*], raccoons [*Procyon lotor*], Wild Turkeys [*Meleagris gallopavo*], nine-banded armadillos [*Dasypus novemcinctus*], and gray foxes [*Urocyon cinereoargenteus*]), and the total number of animal captures on the camera on the respective dependent variable (pathogenic vs any bacteria).

The GEE models were estimated using robust standard errors and an exchangeable working correlation structure. All models were adjusted for the clustering of observations by sampling site, and negative binomial models included the time spent sampling for ticks as an exposure variable. Because wildlife host data was only collected quarterly, all other data was examined on a quarterly basis, e.g. total number of ticks with pathogenic bacteria was calculated for the entire quarter. Quarters were grouped into warm (spring and summer) and cool (fall and winter) seasons due to the fact that trends were homogenous within those groupings as it related to the number of ticks positive for pathogenic/any bacteria.

To create each multivariable model, each variable was examined individually and any variable with a p < 0.2 was included in an initial multivariable model. This initial multivariable model was run and variables with the highest p-value were removed in a step-wise fashion until only variables with a p < 0.1 remained. All variables excluded from this preliminary model were reintroduced one at a time to reassess their significance. After identifying a preliminary main effects model, plausible two-way interactions were evaluated.

## Data Availability

The data from this study are available from the corresponding author upon reasonable request.
